# Resurgence of Persisting Non-Cultivable *Borrelia burgdorferi* following Antibiotic Treatment in Mice

**DOI:** 10.1371/journal.pone.0086907

**Published:** 2014-01-23

**Authors:** Emir Hodzic, Denise Imai, Sunlian Feng, Stephen W. Barthold

**Affiliations:** Center for Comparative Medicine, Schools of Medicine and Veterinary Medicine, University of California Davis, Davis, California, United States of America; University of Toledo School of Medicine, United States of America

## Abstract

The agent of Lyme borreliosis, *Borrelia burgdorferi*, evades host immunity and establishes persistent infections in its varied mammalian hosts. This persistent biology may pose challenges to effective antibiotic treatment. Experimental studies in dogs, mice, and non-human primates have found persistence of *B. burgdorferi* DNA following treatment with a variety of antibiotics, but persisting spirochetes are non-cultivable. Persistence of *B. burgdorferi* DNA has been documented in humans following treatment, but the significance remains unknown. The present study utilized a ceftriaxone treatment regimen in the C3H mouse model that resulted in persistence of non-cultivable *B. burgdorferi* in order to determine their long-term fate, and to examine their effects on the host. Results confirmed previous studies, in which *B. burgdorferi* could not be cultured from tissues, but low copy numbers of *B. burgdorferi flaB* DNA were detectable in tissues at 2, 4 and 8 months after completion of treatment, and the rate of PCR-positive tissues appeared to progressively decline over time. However, there was resurgence of spirochete *flaB* DNA in multiple tissues at 12 months, with *flaB* DNA copy levels nearly equivalent to those found in saline-treated mice. Despite the continued non-cultivable state, RNA transcription of multiple *B. burgdorferi* genes was detected in host tissues, *flaB* DNA was acquired by xenodiagnostic ticks, and spirochetal forms could be visualized within ticks and mouse tissues by immunofluorescence and immunohistochemistry, respectively. A number of host cytokines were up- or down-regulated in tissues of both saline- and antibiotic-treated mice in the absence of histopathology, indicating host response to the presence of non-cultivable, despite the lack of inflammation in tissues.

## Introduction

Experimental animal studies have shown that *Borrelia burgdorferi,* the agent of Lyme borreliosis, consistently establishes persistent infections in a variety of immunocompetent hosts, including laboratory mice [1], white-footed mice (*Peromyscus leucopus)* [2], [3], [4], rats [5], hamsters [6], guinea pigs [7], gerbils [8], dogs [9], and nonhuman primates, including rhesus macaques (*Macaca mulatta)* [10] and baboons (*Papio spp.*) [11]. Clinical evidence extends this paradigm to humans [12]. Persistence is an essential strategy for a complex *B. burgdorferi* life cycle in both ticks and reservoir hosts, and likely pertains to incidental hosts, such as humans. Persistent biology may pose a challenge to antibiotic therapy, though antibiotics ameliorate the majority of host persisting bacteria, by virtue of their immune-evasion biology, may survive in hosts that are unable to clear infection.

Prior to the use of molecular methods to detect spirochetal DNA, treatment of various species of laboratory animals with different classes of antimicrobial drugs has been shown to be successful when the outcome was based upon culture of spirochetes from tissues (reviewed in [13]). With the advent of PCR, and more recently real-time quantitative PCR (qPCR), which offers greater sensitivity and specificity, *B. burgdorferi-*specific DNA has been found to persist for months following antibiotic treatment in tissues of experimentally infected mice [14], [15], [16], [17], [18], [19], [20], dogs [9], [21] and macaques [22], as well as humans [12], [23], [24], [25], [26], [27]. In addition, intact spirochetes have been demonstrated by immunohistochemistry in connective tissue of culture-negative treated mice and within ticks that fed upon them [15], [17] as well as in tissues of and ticks that fed upon treated macaques [22]. Furthermore, RNA transcription of selected *B. burgdorferi* genes has been documented in tissues of treated mice [14] and macaques [22]. *B. burgdorferi* DNA has been shown to be acquired by ticks from treated mice [14], [15], [17] and macaques [22], transmitted by ticks to recipient mice, or transmitted through DNA-positive tissue allografts from treated mice to recipient mice. *B. burgdorferi* DNA was disseminated within the recipient mice, and survived transtadially through molts of larval, nymphal, and adult ticks [14].

Collectively, these studies by multiple research groups in multiple mammalian species following treatment with an array of antimicrobial drugs, including doxycycline, ampicillin, amoxicillin, ceftriaxone, and tigecycline, have documented a similar outcome: persistence of non-cultivable *B. burgdorferi* following antibiotic treatment. One study found that post-treatment persisting non-cultivable spirochetes were genetically attenuated, with loss of one or more plasmids following treatment as an explanation for this unusual state of non-cultivability [15], but this was not confirmed in a subsequent study [17]. It has been conjectured that non-cultivable spirochetes may be inconsequential, since there is no evidence of inflammation in the mice, and that the spirochetes may be in the process of dying, thereby negating their relevance [28]. The Infectious Disease Society of America (IDSA) Guidelines include a statement that “the significance of continued PCR positivity needs to be better understood…” [29]. Clearly, further investigation is needed to resolve these issues.

Treatment of C3H mice with ceftriaxone is a useful model for investigation of post-treatment persistence of non-cultivable *B. burgdorferi*. This strain of mouse is a well-defined model of Lyme borreliosis [30], and treatment of C3H mice with ceftriaxone has been shown by multiple research groups to have consistently resulted in persistence of non-cultivable *B. burgdorferi* following treatment [14], [15], [16], [17], [20]. Therefore, the mouse model allows the opportunity to study the long-term survival or demise of non-cultivable *B. burgdorferi* following treatment, and evaluate tissues for evidence of host response to determine the consequence of their presence.

## Materials and Methods

### Mice

Female C3H/HeN (C3H) mice were purchased from the Frederick Cancer Research Center, Frederick, MD, and inoculated with *B. burgdorferi* at 4–5 weeks of age. Mice were maintained in an isolated room within filter-top cages, and were provided food and water *ad libitum*. All experimental groups of mice, including age-matched uninfected mice, were maintained simultaneously in cohorts of 4 mice per cage under identical husbandry conditions in the same animal room. Mice were killed by carbon dioxide narcosis followed by exsanguination by cardiocentesis.

### Ethics Statement

Animal use was performed under a protocol (#16917) approved by the University of California Davis (UCD) Institutional Animal Care and Use Committee. UCD has a US Public Health Service Animal Welfare Assurance on file and is fully accredited by the Association for the Assessment and Accreditation of Laboratory Animal Care (AAALAC) International. Euthanasia was performed in accordance with guidelines of the American Veterinary Medical Association (AVMA) [31].

### *Borrelia Burgdorferi*

A low-passage clonal strain of *B. burgdorferi* (cN40) was grown in modified Barbour-Stoenner-Kelly (BSKII) medium [32] at 33°C, enumerated under darkfield microscopy with a Petroff-Hausser bacterial counting chamber (Baxter Scientific, McGaw Park, IL), and diluted to appropriate concentrations in BSKII medium. Mice were inoculated subdermally on the dorsal thoracic midline with 10^5^ mid-log phase spirochetes in 0.1 ml of BSKII medium. Mice were inoculated with 10^4^ spirochetes in a confirmatory experiment. Based upon serial dilutions of enumerated *B. burgdorferi* cN40 grown in BSKII medium, culture in BSKII medium is sensitive to a single organism [17], but sensitivity of culture from tissues has not been determined. Infection status with cultivable *B. burgdorferi* was determined by culture of urinary bladder and sub-inoculation site (deep dermis). Based upon over 25 years of experience with this model, these are the most consistent sites for culture in the mouse model. Tissues were collected from mice aseptically at necropsy, and then cultured in medium without antibiotic, as described [1].

### Ticks and Xenodiagnosis

Durland Fish of Yale University provided laboratory-reared, pathogen-free *Ixodes scapularis* larvae. All larvae were derived from single gravid ticks for each study. For xenodiagnosis, 40 larval ticks were placed on each mouse 1 week prior to necropsy, allowed to feed to repletion, collected, and then allowed to molt and harden into nymphs. Cohorts of ticks collected from each mouse were maintained separately, so that ticks within each cohort could be tested individually or in groups by qPCR. Nymphal ticks were frozen in liquid nitrogen, ground with a mortar and pestle, and DNA was extracted for PCR analysis. Nymphal ticks were evaluated in order to demonstrate transstadial persistence of non-cultivable *B. burgdorferi* DNA, rather than direct acquisition of DNA by larvae during their blood meal, and for their larger size to facilitate examination of midgut contents for spirochetal forms.

Selected nymphal ticks were processed by indirect fluorescent antibody (IFA) staining to evaluate for the presence of intact *B. burgdorferi* spirochetes. Individual ticks were dipped in 70% ethanol for 1 min, rinsed in distilled water and then cut longitudinally with a razor blade. Midguts were removed from the ticks and placed in a 10-µl drop of phosphate-buffered saline (PBS) on a glass microscope slide, teased apart and then smeared with micro-forceps. The smears were allowed to dry at room temperature and then fixed in acetone for 10 to 15 min just prior to antibody staining. To block nonspecific binding, slides were incubated with 10% bovine serum albumin (BSA) in PBS at room temperature for 30 min. Following a PBS rinse, the slides were coated with a 1∶10 dilution of anti-*B. burgdorferi* rabbit immune serum for 1 h at 37°C in a humidified chamber. After incubation, slides were washed in PBS 3 times, 1 min each, and then immersed with secondary fluorescein isothiocyanate (FITC) labeled goat anti-rabbit immunoglobulin G (IgG; heavy and light chains). The slides were incubated at 37°C for 30 to 45 min, washed in PBS, dried, and then covered with 90% glycerol-10% PBS and a glass coverslip. Smears were examined with an epifluorescence microscope at 400× magnification with fluorescein emission filters.

### Antibiotic

Mice were administered 16 mg/kg ceftriaxone in 500 µl 0.9% normal saline intraperitoneally twice daily for 5 days and then once daily for 25 days. This dosage regimen was utilized as a model for induction and analysis of persisting non-cultivable *B. burgdorferi*, since it has been previously shown to result in persistence of non-cultivable *B. burgdorferi* when administered at 3–4 weeks or 3–4 months of *B. burgdorferi* N40 infection, and C3H mice were subsequently tested at 1 to 3 months after completion of treatment [14], [15], [16], [17]. Previous studies in this laboratory have shown that the minimal inhibitory concentration (MIC) and minimal bactericidal concentration (MBC) of ceftriaxone is 0.015 µg/ml and 0.06 µg/ml, respectively. Serum ceftriaxone levels were 93 µg/ml, 20 µg/ml, and 2 µg/ml at 1, 2 and 4 hours, respectively, and undetectable at 8 hours in C3H mice following a single injection of 16 mg/kg ceftriaxone, using an agar-based *Staphylococcus aureus* inhibition assay [17]. Thus, peak serum ceftriaxone levels exceeded the *B. burgdorferi* cN40 MIC and MBC [14], [15], [16], [33].

### Tissue Processing for PCR Analysis

Tissue samples for PCR analysis were collected aseptically, immediately weighed, snap-frozen in liquid nitrogen, and stored at −80°C before nucleic acid extraction. For isolation of template DNA for quantitative analysis of spirochetes and total RNA to assess transcriptional activity of selected genes, each sample utilized ∼25 mg of tissue, as described [34]. DNA was extracted from tissues or ticks with DNeasy kits (Qiagen, Valencia, CA) according to the manufacturer’s instructions for tissue or insects, respectively. For analysis of transcriptional activity of selected *B. burgdorferi* and host genes, first-strand cDNA was synthesized from total RNA using the QuantiTect Reverse Transcription Kit (Qiagen), as previously described [35]. The pre-amplified products were diluted at a ratio of 1∶10 and used as templates for PCR analysis. All samples were analyzed for the presence of 18S rRNA in order to determine the efficiency of the nucleic acid extraction, amplification, and as an indicator of inhibition. Tissue samples included the ear, inoculation site, heart base, cardiac ventricular muscle, quadriceps muscle, and left tibiotarsus without overlying skin. These sites represent tissues that become consistently and persistently infected, based upon previous experience [17]. Heart base and tibiotarsus develop transient inflammatory change (carditis and arthritis) during early infection and adjacent tissues (ventricular muscle and quadriceps muscle, respectively) have minimal inflammation [1], [30], [36].

### PCR Analysis

Quantitative real-time PCR (qPCR) was utilized to detect *flaB* DNA in tissue samples and ticks. Approximately 25 mg of each tissue sample was homogenized, DNA was extracted using Qiagen DNeasy kits, the DNA sample was eluted from the column with 200 µl elution buffer, and then the purified DNA/buffer was heated for 5 minutes at 95 degrees. Because low DNA copy numbers were expected in tissues from antibiotic-treated mice, three 5 µl separate aliquots were tested from each 200 µl sample. The analytical sensitivity for the *flaB* gene target was in the range of 1 to 10^9^ copies, as previously described [37]. Each 96-well reaction plate included test DNA samples, positive *B. burgdorferi* cN40 DNA controls, negative reagent controls, and normal mouse tissue DNA controls. Data were summarized as positive or negative, or expressed as the number of DNA copies per mg tissue or per tick.

In order to simultaneously survey for the presence and/or transcription of multiple *B. burgdorferi* and host gene targets, a low density array (LDA) approach was utilized [35]. LDAs were constructed on micro fluidic cards (Applied Biosystems) that contain 8 sample-loading ports, each connected by a micro channel to 48 miniature reaction chambers, for a total of 384 wells per card. Forty-three *B. burgdorferi* cN40 genes were selected from the NCBI Genbank [38]. Genes were selected from different locations throughout the *B. burgdorferi* cN40 genome (chromosome, linear plasmids and circular plasmids). Another LDA was designed to assess transcriptional activity of 19 mouse gene targets, as described [35]. For each of 43 *B. burgdorferi* cN40 and 19 mouse target genes, two specific primers and one internal, fluorescence-labeled probe were designed with Primer Express software (Applied Biosystems), as described [35]. The amplification efficiency (E) of all assays was calculated from the slope of a standard curve generated on a 10-fold dilution in triplicate for every DNA sample, using the formula E = 10^(−1/slope)^ −1. In order to obtain accurate and reproducible results, all assays were determined to have an efficiency of >95%. Based on the amplification efficiencies, detection limits were approximately 10 copies of DNA per reaction. The coefficient of variability of the qPCR determined for 10 replicates was 15% or less. Relative quantification of transcriptional activity of each target gene was calculated using the equation 2^−ΔΔCq^ [39]. C_q_ values of target genes of all samples were normalized to a *B. burgdorferi* reference gene (*16S rRNA*) or mouse reference gene *(β-actin*) to compensate for sample-to-sample and run-to-run variations and to ensure experimental reliability. Additional calculation was performed using Relative Expression Software Tool (REST) [39].

### Histology and Immunohistochemistry

Tissues collected for histopathology included left and right knees, right tibiotarsus, adjacent muscle (quadriceps and others), and hemisected heart (the other half for PCR), including heart base and ventricular muscle. These sites have been determined to be most prone to inflammation, particularly during early infection in the mouse model [1]. Tissues were fixed in neutral buffered formalin, and rear legs were demineralized in acid, embedded in paraffin, sectioned, and stained with hematoxylin and eosin, using standard methods. Tissues were blindly examined for inflammatory lesions. Sections for *B. burgdorferi* immunohistochemistry were processed using polyclonal immune serum from *B. burgdorferi*-infected rabbits (diluted 1∶1,000) and biotinylated goat anti-rabbit immunoglobulin G (Vector Laboratories, Burlingame, CA), as described [40]. Each immunohistochemical batch included negative (by omission of the primary antibody) and positive (tissues from infected C3H*-scid* mice in which spirochetes were previously identified) controls.

### Serology

Antibody titers against *B. burgdorferi* cN40 lysates were determined by enzyme-linked immunosorbent assay (ELISA). Duplicate 3-fold serial dilutions of each serum were incubated with alkaline phosphatase-conjugated rat anti-mouse immunoglobulin (heavy and light chain), as described [41]. Assays included positive and negative control samples. Cutoff points for each serum dilution were based upon the absorbance means of normal mouse serum plus 3 standard deviations (SD) above the means.

### Statistical Analysis

Statistical analysis of qPCR data among treatment groups was performed by one-way analysis of variance, followed by multiple pairwise comparisons by Tukey’s honestly significant difference (HSD) test (SPSS 16.0 for Mac; SPSS Inc., Chicago, IL). Calculated *P* values of less than 0.05 were considered significant.

Statistical analyses of LDA results were conducted with PASW Statistics 18 (IBM). To evaluate the transcriptional level differences of each target gene between collected tissues in mice treated with saline or antibiotic compared to age-matched uninfected C3H mice, one-way ANOVA followed by a least-squares difference post hoc test was performed. Calculated *P* values of less than 0.05 were considered significant. REST © software also tested the transcriptional activity ratio of genes using a pair-wise fixed reallocation randomization test.

REST calculates the relative expression ratios on the basis of group means for target genes versus reference genes, and tests the group ratio results for significance, comparing normalized and not-normalized expression results. The relative and normalized expression ratio was calculated on the basis of the median of the number of technical replicates and computed according to the described equation [39]. This equation contains a correction factor (CF) as well as a multiplication factor (MF). Randomization tests with a pair-wise reallocation were used as the most appropriate approach for this application. They make no assumptions about the distribution of observations in populations. Instead, the software assumes that samples are randomly allocated to control and treatment groups, in keeping with the experimental protocol.

## Results

### Non-cultivable *B. burgdorferi* Resurge in Tissues of Mice at 12 Months Following Antibiotic Treatment

A number of studies by different research groups have shown that C3H mice infected by either syringe or tick, and treated with doxycycline, tigecycline, or ceftriaxone contained persisting non-cultivable *B. burgdorferi* in tissues for at least 90 days after completion of treatment [14], [15], [16], [17], [18], [19], [20]. Others [28] have speculated that the persisting non-cultivable spirochetes did not induce inflammation and would eventually die out following treatment. Thus, a ceftriaxone treatment regimen was used to treat C3H mice similar to previous studies that consistently resulted in persistence of non-cultivable *B. burgdorferi* following treatment [14], [15], [16], [17] to determine the long-term outcome of treatment, including persistence or demise of non-cultivable spirochetes, and returning of cultivability.

Fortyeight 4 week-old C3H mice were infected by inoculation of 10^5^ *B. burgdorferi* cN0. At 30 days of infection, 24 mice were treated with ceftriaxone and 24 mice were sham-treated with saline for 30 days. Groups of 4 antibiotic-treated and 4 sham-treated mice were necropsied at 2 and 4 months after completion of treatment, and 8 antibiotic-treated and 8 sham-treated mice were necropsied at 8 and 12 months after treatment. One week prior to necropsy, 40 larval ticks were placed on each mouse, and collected upon repletion.

Based upon culture of inoculation site and urinary bladder, saline-treated mice were nearly all culture-positive, whereas none of the antibiotic treated mice were culture-positive at any interval after treatment (Table S1). Culture status of saline-treated mice at 4 months was obscured due to contamination of some of the cultures; however, these mice were included based upon their positive PCR status. Based upon *flaB* DNA qPCR on tissues, all mice inoculated with *B. burgdorferi* and treated with saline were *flaB* DNA qPCR-positive at all intervals and in most tissues, except tibiotarsus at 4 and 8 months (Table 1). Because of expected low DNA copy numbers, 3 separate aliquots of each DNA sample from antibiotic-treated mice were tested. Based upon *flaB* DNA qPCR of each set of 3 aliquots, only a few tissue sites were positive at 2, 4 or 8 months in antibiotic-treated mice (Table 2). Among antibiotic-treated mice, 4/4 mice were test positive for *flaB* DNA qPCR at one or more tissue sites at 2 months, 2/4 mice were test positive for *flaB* DNA qPCR at only one tissue site at 4 months, and only 3/8 mice were test positive for *flaB* DNA qPCR at only one tissue site at 8 months. Only 2 tissue sites from 2 mice at the 2 month interval had all three *flaB* DNA qPCR replicates test positive, reflecting the low *flaB* DNA copy numbers in tissues of antibiotic-treated mice compared to saline-treated mice at the first three intervals. Positive samples from antibiotic-treated mice were too sporadic for statistical comparison to samples from saline-treated mice, however, they indeed contained low *flaB* DNA copy numbers. For example, the single tibiotarsus sample in which all 3 aliquots tested positive (+++) from an antibiotic-treated mouse at 2 months contained 12 *flaB* DNA copies (based upon calculation per mg of tissue), compared to a mean of 43±38 SD *flaB* DNA copies among+++tibiotarsus samples from saline-treated mice, and the single+++ventricular muscle sample from an antibiotic-treated mouse contained 37 *flaB* DNA copies, compared to a mean of 225±370 SD *flaB* DNA copies in ventricular muscle of saline-treated mice. Samples in which only 1 or 2 of the triplicate aliquots were positive could only be considered presumptively positive. However, all negative controls tested negative, including 30 additional ear, inoculation site, heart base, ventricular muscle, quadriceps muscle, and tibiotarsus tissue samples from 5 mice inoculated with 10^6^ heat-killed *B. burgdorferi* and tested in triplicate at 3 weeks (data not shown).

**Table 1:** Analysis of *B. burgdorferi flaB* DNA in ear, inoculation site (Inoc), heart base (HB), ventricular muscle (VM), quadriceps muscle (QM), tibiotarsus (Tt), and xenodiagnostic ticks (XenoDx) from mice treated with saline commencing at 30 days after infection.

Interval	Mouse #	Ear	Inoc	HB	VM	QM	Tt	XenoDx
2 months	1	+	+	+	+	+	+	2/2*
	2	+	+	+	+	+	+	3/3
	3	+	+	+	+	+	+	2/3
	4	+	+	+	+	+	+	2/2
4 months	1	+	+	+	+	−	−	0/1
	2	+	+	+	+	+	−	+ (5)**
	3	+	+	+	+	+	−	+ (5)
	4	−	+	+	+	+	−	+ (5)
8 months	1	−	−	+	+	−	−	+ (5)
	2	+	−	+	+	+	−	+ (5)
	3	−	−	+	+	+	−	2/2
	4	−	+	+	+	+	+	2/3
	5	−	−	+	+	+	−	+ (5)
	6	+	+	+	+	+	−	2/2
	7	+	−	+	+	+	+	2/3
	8	−	−	−	+	+	−	+ (5)
12 months	1	+	+	+	+	+	+	3/5
	2	+	+	+	+	+	+	4/5
	3	+	+	+	+	+	−	2/5
	4	+	+	+	+	+	+	5/5
	5	−	+	+	+	+	+	0/5
	6	−	+	+	+	−	+	4/5
	7	−	+	+	+	+	+	1/5
	8	+	+	+	+	+	+	2/5

Mice were tested at 2, 4, 8, or 12 months after completion of treatment.

* number of positive ticks/number of ticks tested.

** +(5) = result of a pooled sample from 5 ticks.

**Table 2:** Analysis of *flaB* DNA in ear, inoculation site (Inoc), heart base (HB), ventricular muscle (VM), quadriceps muscle (QM), tibiotarsus (Tt), and xenodiagnostic ticks (XenoDx) from mice treated with ceftriaxone commencing at 30 days after inoculation.

Interval	Mouse #	Ear	Inoc	HB	VM	QM	Tt	XenoDx
2 months	1	−−−	−−−	−−−	−−−	−−−	+++	0/3*
	2	+−−	−−−	++−	−−−	−−−	−−−	0/4
	3	−−−	−−−	−−−	+++	−−−	+−−	0/2
	4	+−−	−−−	−−−	−−−	−−−	+−−	0/2
4 months	1	−−−	−−−	−−−	−−−	−−−	−−−	ND
	2	−−−	−−−	−−−	−−−	−−−	−−−	ND
	3	−−−	−−−	+−−	−−−	−−−	−−−	0/3
	4	−−−	−−−	−−−	−−−	−−−	++−	ND
8 months	1	−−−	−−−	−−−	−−−	−−−	+−−	ND
	2	−−−	−−−	−−−	−−−	−−−	−−−	3/4
	3	−−−	−−−	−−−	−−−	−−−	−−−	0/2
	4	−−−	−−−	−−−	−−−	−−−	−−−	ND
	5	−−−	−−−	−−−	+−−	−−−	−−−	0/3
	6	−−−	−−−	−−−	−−−	−−−	+−−	1/6
	7	−−−	−−−	−−−	−−−	−−−	−−−	1/3
	8	−−−	−−−	−−−	−−−	−−−	−−−	2/2
12 months	1	−−−	+−−	+++	+++	++−	+−−	0/10
	2	−−−	+−−	+++	+−−	+++	−−−	1/10
	3	++−	−−−	+++	+++	++−	+++	1/10
	4	+−−	+++	++−	+−−	++−	+−−	1/10
	5	−−−	+++	+++	+++	+++	−−−	1/10
	6	+++	+++	+++	+−−	+++	+++	0/10
	7	−−−	+++	+++	+++	+++	+++	1/10
	8	+++	+++	+++	+++	+++	+++	1/10

Mice were tested at 2, 4, 8, or 12 months after completion of treatment. Three separate aliquots of each tissue homogenate were tested, and positive or negative PCR results for each aliquot are summarized for each tissue.

* number of positive ticks/number of ticks tested.

**Figure 1:**
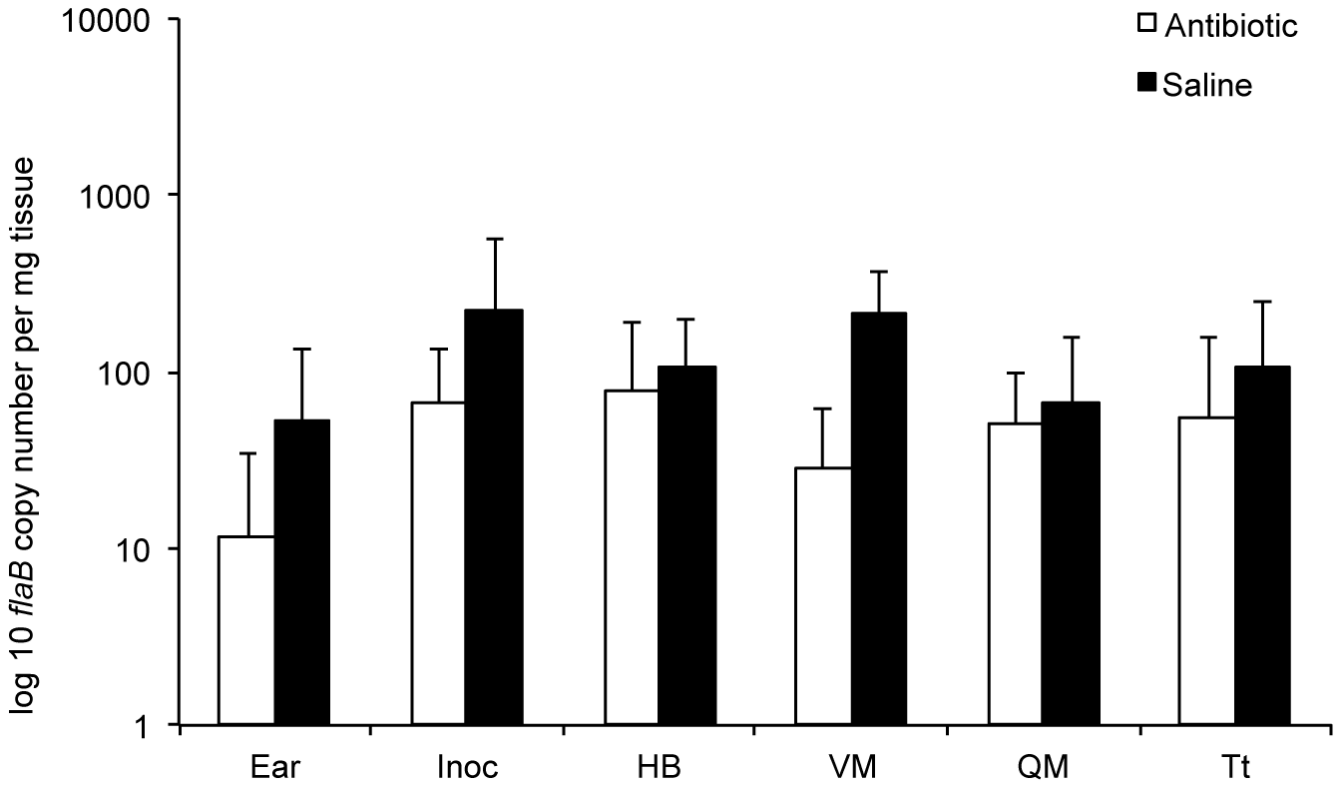
*Borrelia burgdorferi* levels resurge in tissues at 12 months after antibiotic treatment. Copy numbers of *B. burgdorferi flaB* DNA, determined by quantitative PCR, in ear, inoculation site (Inoc), heart base (HB), ventricular muscle (VM), quadriceps muscle (QM), and tibiotarsus (Tt) tissues of saline- and antibiotic-treated mice at 12 months after treatment.

In contrast to the first 3 intervals, all antibiotic−treated mice and all saline-treated mice at the 12 month interval were test positive for *flaB* DNA qPCR at multiple tissue sites, and in most cases, all 3 triplicate samples were *flaB* DNA qPCR-positive (Table 2). In addition, *flaB* DNA copy numbers in all tissues of antibiotic-treated mice were nearly equivalent to *flaB* DNA copy numbers in respective tissues of saline-treated mice at 12 months (*P*<0.05) (Figure 1). The validity of these findings was confirmed by randomizing 8 negative samples from antibiotic-treated mice at 2, 4, and 8 months, plus 8 positive samples from antibiotic-treated mice at 12 months, and blindly retesting them in triplicate along with positive and negative controls. The results were identical compared to previous testing. Thus, these results suggested resurgence of spirochetes at 12 months following antibiotic treatment, although all mice remained culture-negative at all intervals.

**Figure 2:**
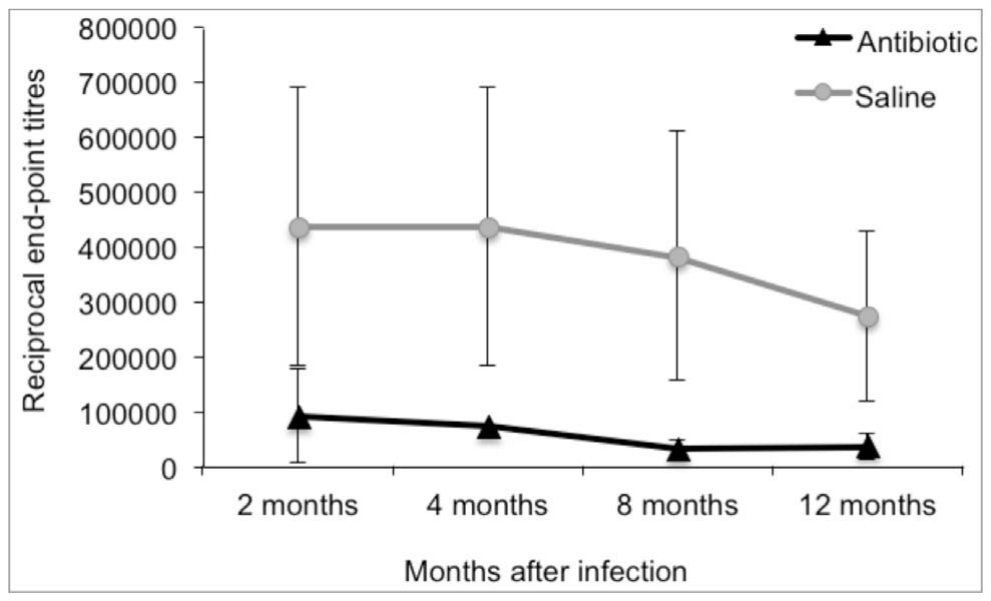
Antibody titers to *Borrelia burgdorferi* are significantly lower following antibiotic treatment, and do not rise in response to resurgence at 12 months. Reciprocal end-point dilutions of sera reacted against *B. burgdorferi* lysate antigen.

Serum antibody reactivity to *B. burgdorferi* lysate antigen was significantly lower in antibiotic-treated mice compared to saline-treated mice at 2 months after completion of treatment (Figure 2). Antibody titers declined with time in both groups, with no evidence of an increase in antibiotic-treated mice at 8 or 12 months in spite of the resurgence of spirochete burdens in tissues at 12 months.

### Detection of Persisting *B. burgdorferi* in Xenodiagnostic Ticks that Fed upon Antibiotic-treated Mice

**Figure 3:**
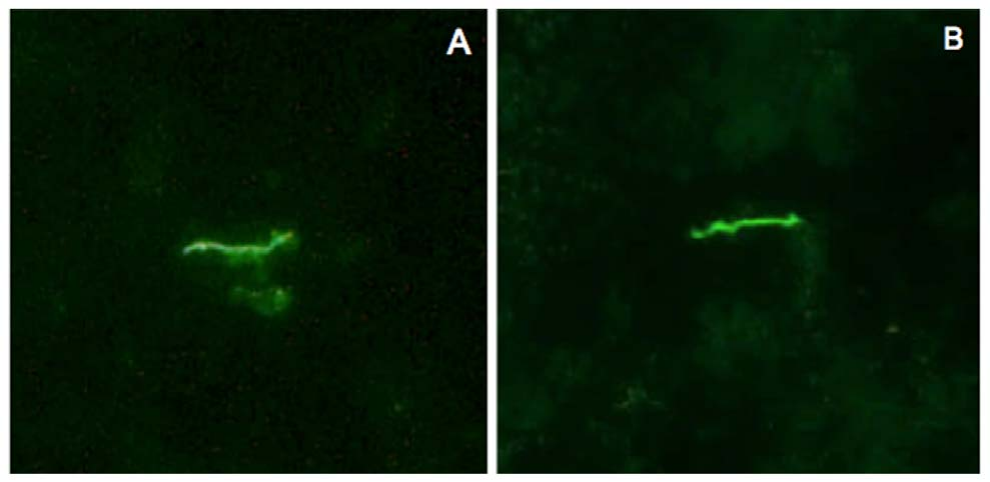
Spirochetes are present within xenodiagnostic ticks that fed upon saline- or antibiotic-treated mice at 12 months after treatment. Indirect immunofluorescent staining of *B. burgdorferi* in the midguts of ticks that fed upon saline-treated (A) or ceftriaxone-treated mice (B) at 12 months after treatment.

Xenodiagnosis is a highly sensitive method for detecting *B. burgdorferi* infection, reflecting acquisition of spirochetes by feeding ticks [3]. Larval ticks were fed to repletion on each mouse, and allowed to molt and harden into nymphs. Surviving nymphs within each cohort that fed upon each mouse were tested by qPCR. Xenodiagnosis results for ticks that fed upon infected mice at 2, 8 and 12 months after antibiotic or saline treatment are summarized (Tables 1 and 2). Ticks were PCR-positive at all intervals in both treatment groups. At 12 months, despite relatively similar *flaB* DNA copy numbers in tissues of both antibiotic- and saline-treated mice, *flaB* DNA copy numbers were significantly lower in ticks that fed upon antibiotic-treated mice (mean 528±1,002 SD) compared to ticks that fed upon saline-treated mice (mean 2,590±4,450 SD) (*P* = 0.044). The lower *flaB* DNA copy numbers in ticks that fed upon antibiotic-treated mice reflected either a lower capacity to replicate within ticks, reduced transstadial persistence of DNA, or acquisition of a lower number of spirochetes during feeding. However, these results nevertheless indicated that mice treated with antibiotics were xenodiagnosis DNA-positive. In addition, selected ticks that fed upon infected saline-treated mice and antibiotic-treated mice at 12 months were dissected and examined by fluorescence microscopy for antibody-reactive spirochetes. Immunofluorescent spirochetes were found in ticks that fed upon both infected saline- and antibiotic-treated mice (Figure 3). Thus, these results confirmed the presence of intact *B. burgdorferi* spirochetes and spirochetal DNA in xenodiagnostic ticks that fed upon both infected saline-treated and antibiotic-treated mice at 12 months following treatment.

### RNA Transcription of Host Genes in Response to Non-cultivable *B. burgdorferi*

**Figure 4:**
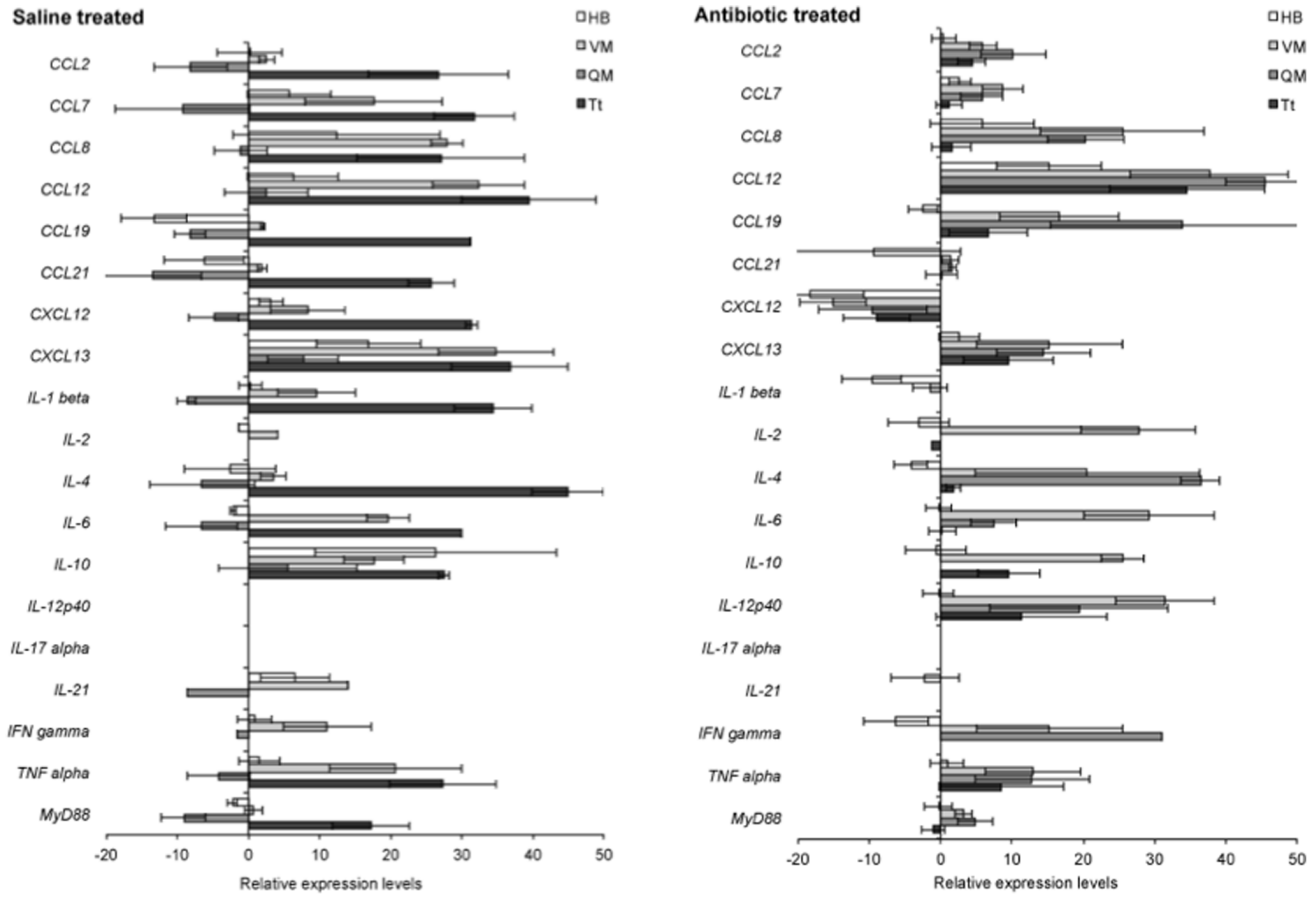
Persisting *B. burgdorferi* elicit host cytokine responses at 12 months following saline or antibiotic treatment. Transcription of 19 host cytokine cDNAs, relative to uninfected, age-matched control mice in heart base (HB), ventricular muscle (VM), quadriceps muscle (QM) and tibiotarsus (Tt) at 12 months after treatment with saline or antibiotic.

The relative levels of host cytokine gene transcription was evaluated by the LDA approach in heart base and tibiotarsus tissues of saline-treated infected mice and antibiotic-treated mice at 12 months after treatment, compared to gene transcription in tissues of uninfected, age-matched mice maintained under the same environmental conditions. A number of host cytokine genes were significantly up- or down-regulated in both saline- and antibiotic-treated mice at 12 months after treatment, compared to uninfected, age-matched controls (Figure 4, Table S2). Conclusions regarding up- or down-regulation of specific genes cannot be made with these data, but the results implied that host cytokines were variably active or suppressed in tissues of infected saline- and antibiotic-treated mice at 12 months after treatment relative to tissues of uninfected, age-matched control mice.

### Confirmation of Resurgence of Non-cultivable Spirochetes at 12 Months

In order to confirm the resurgence phenomenon, a repeat experiment was performed (Table 3). Sixty C3H mice were inoculated with 10^4^ *B. burgdorferi,* which was one log less than the first experiment. Antibiotic or saline treatment did not commence until 42 days of infection (in contrast to 30 days in the first experiment). Ten antibiotic-treated infected mice were sampled at 2, 4, 8, and 12 months, and 5 saline-treated infected and 5 saline-treated uninfected mice were sampled at 2, 4, 8, and 12 months. As before, none of the 40 antibiotic-treated mice at any interval was culture-positive, whereas 5/5, 4/4, 5/5, and 4/5 saline-treated infected mice were culture-positive at 2, 4, 8, and 12 months, respectively. Among antibiotic-treated mice, 6/10 mice at 2 months after treatment were tested *flaB* DNA-positive at one or more tissue sites, 7/10 mice were *flaB* DNA-positive at 4 months, and only 1/10 mice had a single tissue site that was *flaB* DNA-positive at 8 months. At 12 months after antibiotic treatment, 8/10 antibiotic-treated mice had one or more *flaB* DNA-positive tissues, particularly tibiotarsus. Copy numbers of *flaB* DNA within positive tibiotarsus tissues from antibiotic-treated mice at 12 months were lower (mean 29±23 SD) than in saline-treated mice (mean 766±1,600 SD) but differences were not significantly different (P = 0.1213). Although resurgence did not occur in as many tissues of antibiotic-treated mice as the first experiment, results of this repeat study confirmed resurgence of non-cultivable *B. burgdorferi* at 12 months following treatment. The differences in the two experiments may be related to the initial infecting dose of spirochetes and the older age of the mice at the time of treatment, both of which influence infection kinetics of early infection [30].

**Table 3:** Table **3.** Analysis of *flaB* DNA in ear, inoculation site (Inoc), heart base (HB), ventricular muscle (VM), quadriceps muscle (QM), and tibiotarsus (Tt) from mice treated with ceftriaxone commencing at 42 days of infection.

Interval	Mouse #	Ear	Inoc	HB	VM	QM	Tt
2 months	1	−−−	−−−	−−−	−−−	−−−	−−−
	2	−−−	+−−	−−−	−−−	−−−	−−−
	3	−−−	−−−	−−−	+−−	−−−	+−−
	4	−−−	−−−	−−−	+−−	−−−	−−−
	5	−−−	−−−	−−−	−−−	−−−	−−−
	6	−−−	−−−	−−−	−−−	−−−	+−−
	7	−−−	−−−	−−−	+−−	−−−	−−−
	8	−−−	−−−	+−−	−−−	−−−	−−−
	9	−−−	−−−	−−−	−−−	−−−	−−−
	10	−−−	−−−	−−−	−−−	−−−	−−−
4 months	1	−−−	−−−	−−−	−−−	−−−	+−−
	2	−−−	−−−	−−−	−−−	−−−	+−−
	3	−−−	−−−	−−−	−−−	−−−	−−−
	4	−−−	−−−	−−−	−−−	−−−	−−−
	5	−−−	−−−	−−−	−−−	−−−	+−−
	6	−−−	−−−	+−−	−−−	−−−	++−
	7	−−−	−−−	−−−	−−−	−−−	+−−
	8	−−−	−−−	−−−	−−−	−−−	+−−
	9	−−−	−−−	−−−	−−−	−−−	−−−
	10	−−−	−−−	−−−	−−−	−−−	+−−
8 months	1	−−−	−−−	−−−	−−−	−−−	−−−
	2	−−−	−−−	+−−	−−−	−−−	−−−
	3	−−−	−−−	−−−	−−−	−−−	−−−
	4	−−−	−−−	−−−	−−−	−−−	−−−
	5	−−−	−−−	−−−	−−−	−−−	−−−
	6	−−−	−−−	−−−	−−−	−−−	−−−
	7	−−−	−−−	−−−	−−−	−−−	−−−
	8	−−−	−−−	−−−	−−−	−−−	−−−
	9	−−−	−−−	−−−	−−−	−−−	−−−
	10	−−−	−−−	−−−	−−−	−−−	−−−
12 months	1	−−−	−−−	+−−	−−−	−−−	+++
	2	−−−	+−−	+++	−−−	−−−	+−−
	3	−−−	−−−	−−−	−−−	−−−	−−−
	4	−−−	−−−	−−−	−−−	−−−	−−−
	5	−−−	−−−	−−−	−−−	−−−	+++
	6	+−−	−−−	−−−	−−−	−−−	−−−
	7	−−−	−−−	−−−	−−−	−−−	+++
	8	−−−	−−−	+−−	−−−	−−−	+++
	9	−−−	−−−	−−−	−−−	−−−	++−
	10	−−−	−−−	−−−	−−−	−−−	+++

Mice were tested at 2, 4, 8, or 12 months after completion of treatment. Three separate aliquots of each tissue homogenate were tested, and positive or negative PCR results for each aliquot are summarized for each tissue.

### RNA Transcription by Persisting Non-cultivable *B. burgdorferi*

Tissue samples from the repeat experiment were processed for cDNA in order to evaluate RNA transcription by non-cultivable spirochetes (Table S3). The LDA approach lent itself to simultaneous interrogation of transcription for 43 different *B. burgdorferi* genes. Among tissue samples from antibiotic-treated mice, there were 3 *flaB* DNA-positive heart base samples and 7 positive tibiotarsus samples, which were processed for LDA analysis of cDNA. In addition, *flaB* DNA-positive heart base and tibiotarsus from all 5 saline-treated infected mice were processed for comparison. Samples were assayed in duplicate. All *flaB* DNA-positive tissue samples from both treatment groups were *B. burgdorferi* 16S ribosomal cDNA-positive. Among the other 42 gene targets tested, representing genes located within the chromosome, linear plasmids and circular plasmids, gene transcription was detected in both duplicate samples of 22 gene targets in saline-treated mice, particularly in heart base samples. Among those 22 positive gene targets in the saline-treated mice, duplicate samples of 18 gene targets tested positive in tissues from 1 or more antibiotic-treated mice. These results indicated that persisting non-cultivable *B. burgdorferi* that resurged at 12 months were transcribing multiple *B. burgdorferi* genes. Due to low copy numbers of cDNA and the lower sensitivity for detection of cDNA, further conclusions regarding specific gene transcription could not be made.

### Histopathology and Immunohistochemistry of Persisting Non-cultivable *B. burgdorferi* in Tissues of Mice

Tissues were not collected for histopathology in the first experiment, since inflammatory lesions are typically minimal or absent during persistent infection in untreated infected mice, in spite of the continued presence of spirochetes in tissues [1], [30]. Since resurgence of non-cultivable *B. burgdorferi* at 12 months was found in the first experiment, heart and leg (knee, tibiotarsus and muscle) were collected and examined for histopathology at the 12 month post-treatment interval in the confirmatory experiment. No inflammation (arthritis) was found in joints of infected saline-treated mice, but all infected saline-treated mice had segmental lymphocytic infiltration of the adventitia of plantar arteries, and mild lymphocytic infiltrates in the epicardium and periaortic adventitia in the heart base, as previously described [1], [30]. Heart tissue of the single infected, antibiotic-treated mouse with +++ *flaB* DNA were shown sparse lymphocytic infiltrates in the epicardium at the base of the heart, and another antibiotic-treated mouse had a small focus of segmental lymphocytic infiltration in the adventitia of a plantar artery. Despite the prevalence of *flaB* DNA in tibiotarsal tissue in both treatment groups, there was no evidence of joint inflammation (arthritis) or carditis in either group.

**Figure 5:**
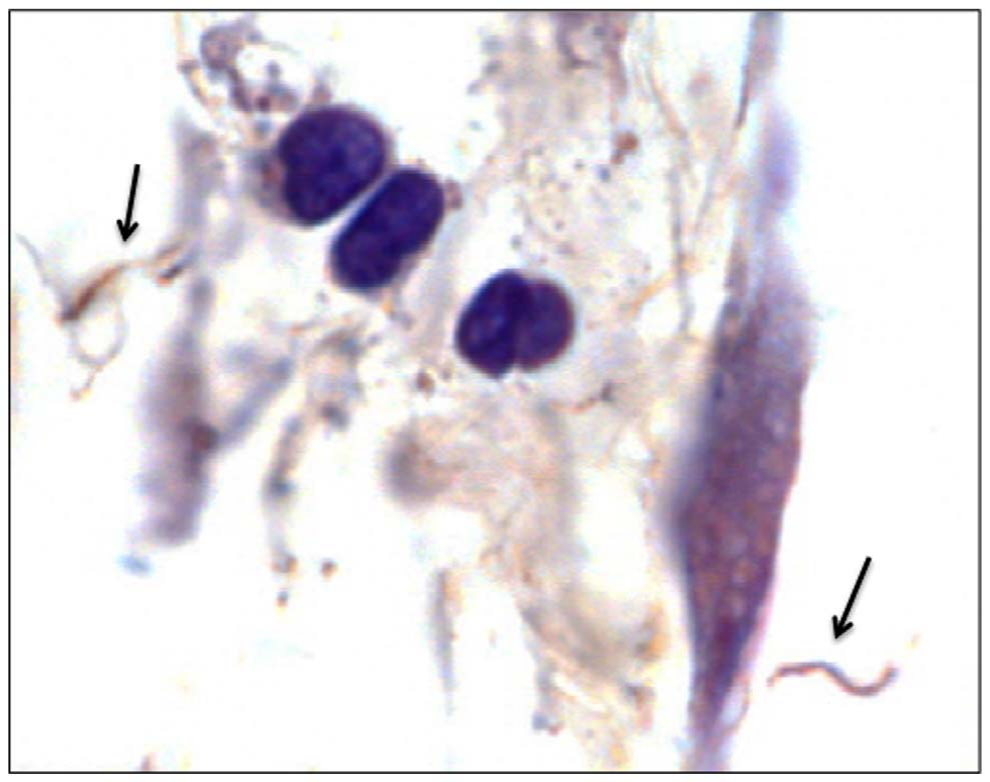
Spirochetes can be visualized within tissue of a mouse at 12 months following antibiotic treatment. Indirect immunohistochemical staining of *B. burgdorferi* spirochetes (arrows) in the heart base connective tissue.

DNA-positive heart tissue from the+++antibiotic-treated mouse was processed for immunohistochemistry. Multiple but rare spirochetes were visualized in the extracellular matrix of connective tissue in the heart base and epicardium (Figure 5). Notably, when found, multiple extracellular spirochetes co-localized within the connective tissue of affected areas, but most were visible in and out of the Z plane of focus (could not be photographed in their entirety). Further analysis of additional tissues was not performed, due to the extreme difficulty in finding spirochetes, even in tissues of untreated, persistently infected mice. These results confirmed that morphologically intact, antigen-reactive spirochetal forms were present in cardiac connective tissue of an infected antibiotic-treated mouse at 12 months after treatment.

## Discussion

This study validates a mouse model that can be used for investigation of post-antibiotic persistence of non-cultivable *B. burgdorferi.* We utilized syringe-inoculation of cultured *B. burgdorferi*, which is not the natural route of infection, but others [15], [16] have shown persistence of non-cultivable *B. burgdorferi* with tick-borne infection as well. Animal-based studies, particularly those performed in mice, have been challenged because the pharmacokinetics of ceftriaxone in the mouse differ from those in humans [28]. Although the treatment regimen used in this and other studies achieved more than adequate serum MIC and MBC levels, the serum half-life for ceftriaxone was only 1.1 hours in mice [42]. The current study was not focused on mimicking a treatment regimen equivalent to that used in humans. The experimental approach utilized a ceftriaxone regimen used by multiple research groups that consistently resulted in persistence of non-cultivable *B. burgdorferi* following treatment in C3H mice in order to determine the ultimate fate, up to 12 months, of persisting non-cultivable spirochetes. Furthermore, the short serum half-life of ceftriaxone in mice as an explanation for persistence of non-cultivable *B. burgdorferi* was negated with a study that utilized tigecycline [14], which has a half-life of 11.6 hours in mice and achieved a serum maximum level 70 times greater than the *B. burgdorferi* N40 MBC [43]. Treatment with ceftriaxone, and high or low doses of tigecycline resulted in the same outcome: persistence of non-cultivable *B. burgdorferi* in tissues of treated mice at 90 days after completion of treatment [14].

Results of this study demonstrated not only persistence, but also resurgence of non-cultivable *B. burgdorferi* in tissues of mice at up to 12 months following antibiotic treatment, despite the continued inability to culture spirochetes from tissues. In the absence of the ability to culture spirochetes from tissues of treated mice, this study endeavored to determine the physiologic state of persisting non-cultivable *B. burgdorferi.* Non-cultivable *B. burgdorferi* DNA acquisition was demonstrated by xenodiagnosis (including transstadial persistence from larva to nymph), *B. burgdorferi* spirochetal forms were visualized by immunofluorescence in xenodiagnostic ticks, RNA transcription of multiple *B. burgdorferi-*specific genes was detected in the tissues of antibiotic-treated mice at 12 months after antibiotic treatment, and spirochetal forms were visualized in the tissue of an antibiotic-treated mouse by immunohistochemistry. It has been speculated that following antibiotic treatment, persisting non-cultivable spirochetes were non-viable and would eventually be eliminated [15], [28], [44]. Results of the current study suggested a similar trend if the experiment were to be stopped at 8 months, as the prevalence of PCR-positive tissue sites declined with time through 8 months. However, there was resurgence of *B. burgdorferi* DNA in tissues at 12 months to levels nearly equivalent to those in untreated persistently infected mice. These findings underscore the difficulty of detecting non-cultivable *B. burgdorferi* following antibiotic treatment, as interpretation of xenodiagnosis depends upon timing and number of ticks tested, and the outcome of infection must be measured by analysis of multiple tissues at appropriate intervals. Non-cultivability of persisting *B. burgdorferi* is likely a reflection of their limited replication kinetics. In a recent study involving antibiotic-treated macaques, small numbers of spirochetes were found in culture after several weeks of incubation [22], and others have demonstrated slow outgrowth of spirochetes in cultures from antibiotic-treated humans after prolonged incubation of up to 10.5 months [45]. In the present study, we attempted to incubate cultures for several weeks, as in the macaque study, but were unable to grow viable spirochetes.

The mechanism for resurgence of non-cultivable *B. burgdorferi* at 12 months after treatment remains to be determined, but may be related to declining antibody response. Antibody is critical for resolution of arthritis and carditis, as well as global reduction of spirochete burdens in tissues [40], [46], [47]. Disease resolution can be maintained and reduced tissue burdens can be sustained in infected severe combined immunodeficient mice by passive immunization with immune serum from persistently infected immunocompetent mice, but disease recurs and resurgence of spirochete burdens occurs as the passive antibody decays [48]. The antigenic target for maintaining reduced tissue burdens has not been determined, so cause and effect in the current study could not be confirmed. Antibody titers against several *in vivo*-expressed N40 recombinant proteins (DbpA, OspC, Arp, BmpA) [49], similar to antibody against *B. burgdorferi* lysates, were also diminished at all intervals following antibiotic treatment (data not shown). In a related study, recrudescence of non-cultivable *B. burgdorferi* following antibiotic treatment was attempted by transient immunosuppression of the mice with corticosteroids without apparent consequence [15], but that outcome would be expected when spirochete burdens in tissues are under control of antibody.

The current study builds upon similar evidence of non-cultivable *B. burgdorferi* persistence in studies involving dogs, mice and macaques. While it has been concluded that non-cultivable *B. burgdorferi* is not infectious [16], we previously demonstrated transmission of *B. burgdorferi* DNA by ticks that fed upon antibiotic-treated mice to naïve mice, as well as transmission of *B. burgdorferi* DNA by transplantation of *B. burgdorferi* DNA-positive heart base and tibiotarsus tissue allografts from treated mice into recipient mice, with dissemination of the DNA in the recipient mice [14]. Transtadial transmission of *B. burgdorferi* DNA from larvae that fed upon treated mice to nymphs and then to adults was also demonstrated [14]. RNA transcription of a limited number of *B. burgdorferi* genes by persisting non-cultivable spirochetes was shown after antibiotic treatment of both mice [14] and macaques [22], but analyses were limited to only a few target genes. In the current study, the LDA approach allowed simultaneous analysis of multiple gene targets, indicating more global transcription activity of persisting non-cultivable *B. burgdorferi* at 12 months. Added to earlier studies that demonstrated persistence of *B. burgdorferi* DNA in tissues of antibiotic-treated dogs [9], [21], [50], the collective conclusion is persistence of non-cultivable spirochetes following treatment of mice, macaques and dogs with various antibiotics. The limited infectivity of persisting non-cultivable *B. burgdorferi* has no biological significance for the natural enzootic cycle of *B. burgdorferi,* but it remains to be determined if they have significance in the post-treatment host. Thus, validation of a reproducible animal model for study of this phenomenon is important.

It has been suggested that persisting non-cultivable *B. burgdorferi* may be irrelevant, in that they are non-pathogenic and do not cause disease [28]. This is difficult to prove, since in the normal course of events, fully virulent *B. burgdorferi* infects multiple tissues without inflammation. Experimental animal studies have shown that arthritis and carditis are more severe in animals that are genetically susceptible, immature or immunodeficient, or in animals inoculated with high doses of spirochetes that can replicate to high enough titer to colonize specific tissue sites before immune-mediated disease remission [30]. Inflammation is related to colonization of specific tissue sites that are prone to inflammation, including synovium and vascular media, resulting in acute arthritis (synovitis), arteritis and carditis. The host antibody response eliminates or prevents colonization of these sites, so that the normal course of infection in adult, immunocompetent animals is minimal or none. The lack of inflammation, therefore, in tissues of antibiotic-treated mice at 12 months, with resurgence of spirochete DNA burdens equivalent to those found in saline-treated mice, does not prove the argument one way or the other, since there is no or minimal inflammation in tissues of infected saline-treated mice at 12 months with similar or greater spirochete burdens. The only histologic evidence of host response in both groups of mice was segmental lymphoplasmacytic infiltrates in plantar artery adventitia and sparse infiltrates in the epicardium, albeit less obvious in the antibiotic-treated mice.

The lack of morphological evidence of inflammation under normal circumstances, as well as after antibiotic treatment, prompted us to evaluate host response through LDA analysis of host cytokines. Avoiding over-interpretation of specific cytokine responses, our results indicated that mice were responding to the presence of persisting non-cultivable *B. burgdorferi.* The LDA approach allows simultaneous analysis of multiple host cytokines relative to age-matched uninfected mouse tissues, but does not measure absolute values. It is therefore useful for visualizing a “fingerprint” of host response, indicating a pro-inflammatory cytokine state in both persistently infected, saline-treated and antibiotic-treated mice at 12 months after treatment. Recently, it has been shown that spirochete antigens persist in tissues of antibiotic-treated mice, which may be responsible for cytokine activation [16]. The presence of persisting, non-cultivable *B. burgdorferi* that transcribe RNA and express antigens in tissues may contribute to this phenomenon.

Because of the controversial nature of these findings, they should not be over-interpreted and certainly not translated directly into clinical management of human Lyme borreliosis. However, as in various animal studies, persisting *B. burgdorferi-*specific DNA has been documented following antibiotic treatment in human Lyme borreliosis [12], [23], [24], [25], [26], [27].

Although various animal studies may each have their flaws (as do human clinical studies), the comparative evidence in dogs, mice, non-human primates, and perhaps humans, is compelling, and suggests that something unique is happening with *B. burgdorferi* following antibiotic treatment. This study validates a reproducible model for studying this phenomenon.

## Supporting Information

Table S1 ***Borrelia*** **burgdorferi culture results (number positive/number tested) of inoculation sites and urinary bladders of mice treated with saline or ceftriaxone commencing at 30 days of infection, and then cultured at 2, 4, 8 or 12 months following completion of treatment.**
(DOCX)

Table S2 **Statistical difference probability values of various cytokine cDNA levels in tissues of** ***Borrelia burgdorferi*** **- infected mice at 12 months following treatment with antibiotic or saline, relative to age-matched uninfected mice.**
(DOCX)

Table S3 **LDA analysis for RNA transcription of selected genes located throughout the** ***B. burgdorferi*** **cN40 genome in** ***flaB*** **DNA-positive heart base (HB) and tibiotarsal (Tt) tissue samples from infected mice at 12 months following treatment with saline or antibiotic.**
(DOCX)
