# Supplementary material for: Resurgence of Persisting Non-Cultivable Borrelia burgdorferi following Antibiotic Treatment in Mice
Source: PLoS One. 2014 Jan 23;9(1):e86907. doi: 10.1371/journal.pone.0086907 (PMC3900665; doi:10.1371/journal.pone.0086907)
Supplement: Table S1 — Borrelia burgdorferi culture results (number positive/number tested) of inoculation sites and urinary bladders of mice treated with saline or ceftriaxone commencing at 30 days of infection, and then cultured at 2, 4, 8 or 12 months following completion of treatment. (DOCX) [file pone.0086907.s001.docx]

**Table S1.** *Borrelia* burgdorferi culture results (number positive/number tested) of inoculation sites and urinary bladders of mice treated with saline or ceftriaxone commencing at 30 days of infection, and then cultured at 2, 4, 8 or 12 months following completion of treatment.

| **Treatment** | **Interval** | **Urinary Bladder** | **Inoculation Site** | **Combined** |
| --- | --- | --- | --- | --- |
| Ceftriaxone | 2 months | 0/4 | 0/4 | 0/4 |
|  | 4 months | 0/4 | 0/4 | 0/4 |
|  | 8 months | 0/8 | 0/8 | 0/8 |
|  | 12 months | 0/7* | 0/8 | 0/8 |
| Saline | 2 months | 3/3* | 4/4 | 4/4 |
|  | 4 months | 3/4* | 1/1* | 3/4 |
|  | 8 months | 7/8 | 5/6* | 8/8 |
|  | 12 months | 4/8 | 5/8 | 7/8 |

* one or more samples contaminated.
