# Supplementary material for: Resurgence of Persisting Non-Cultivable Borrelia burgdorferi following Antibiotic Treatment in Mice
Source: PLoS One. 2014 Jan 23;9(1):e86907. doi: 10.1371/journal.pone.0086907 (PMC3900665; doi:10.1371/journal.pone.0086907)
Supplement: Table S2 — Statistical difference probability values of various cytokine cDNA levels in tissues of Borrelia burgdorferi - infected mice at 12 months following treatment with antibiotic or saline, relative to age-matched uninfected mice. (DOCX) [file pone.0086907.s002.docx]

**Table S2.**  Statistical difference probability values of various cytokine cDNA levels in tissues of *Borrelia burgdorferi*- infected mice at 12 months following treatment with antibiotic or saline, relative to age-matched uninfected mice.

| **Treatment** | **Tissue** | **Cytokine*** | **Up or Down** | ***P* value**** |
| --- | --- | --- | --- | --- |
| Antibiotic | Heart base | CCL12 | up | 0.0004 |
|  |  | CCL21 | down | 0.024 |
|  |  | CXCL12 | down | 0.001 |
|  |  | IL1b | down | 0.023 |
|  | Ventricular muscle | CCL2 | up | 0.001 |
|  |  | CCL7 | up | 0.004 |
|  |  | CCL8 | up | 0.001 |
|  |  | CCL12 | up | 0.001 |
|  |  | CCL19 | up | 0.002 |
|  |  | CXCL12 | down | 0.001 |
|  |  | CXCL13 | up | 0.001 |
|  |  | IL1b | down | 0.018 |
|  |  | IL2 | up | 0.002 |
|  |  | IL6 | up | 0.001 |
|  |  | IL10 | up | 0.047 |
|  |  | IL12p40 | up | 0.0001 |
|  |  | INFγ | up | 0.04 |
|  |  | MyD88 | up | 0.002 |
|  | Tibiotarsus | CCL2 | up | 0.0001 |
|  |  | CCL12 | up | 0.002 |
|  |  | CXCL12 | down | 0.002 |
|  | Quadriceps muscle | CCL2 | up | 0.027 |
|  |  | CCL7 | up | 0.002 |
|  |  | CCL8 | up | 0.001 |
|  |  | CCL12 | up | 0.001 |
|  |  | CCL19 | up | 0.001 |
|  |  | CCL21 | up | 0.021 |
|  |  | CXCL12 | down | 0.001 |
|  |  | IL4 | up | 0.008 |
|  |  | IL6 | up | 0.026 |
|  |  | IL12p40 | up | 0.004 |
|  |  | TNFα | up | 0.001 |
|  |  | MyD88 | up | 0.001 |
| Saline | Heart base | CCL7 | up | 0.001 |
|  |  | CCL8 | up | 0.028 |
|  |  | CCL19 | down | 0.017 |
|  |  | CCL21 | down | 0.013 |
|  |  | CXCL13 | up | 0.025 |
|  |  | IL10 | up | 0.003 |
|  |  | IL21 | up | 0.025 |
|  | Ventricular muscle | CCL2 | up | 0.01 |
|  |  | CCL7 | up | 0.007 |
|  |  | CCL8 | up | 0.002 |
|  |  | CCL12 | up | 0.002 |
|  |  | CXC12 | down | 0.007 |
|  |  | CXCL13 | up | 0.017 |
|  |  | IL1b | up | 0.001 |
|  |  | IL6 | up | 0.015 |
|  |  | IL10 | up | 0.003 |
|  |  | INFγ | up | 0.039 |
|  |  | TNFα | up | 0.003 |
|  | Tibiotarsus | CCL2 | up | 0.043 |
|  |  | CCL7 | up | 0.021 |
|  |  | CCL12 | up | 0.009 |
|  |  | CXCL12 | up | 0.005 |
|  | Quadriceps muscle | CCL7 | down | 0.001 |
|  |  | CCL19 | down | 0.032 |
|  |  | CCL21 | down | 0.001 |
|  |  | CXCL12 | down | 0.001 |
|  |  | CXCL13 | up | 0.005 |
|  |  | IL1b | down | 0.001 |
|  |  | IL6 | down | 0.026 |
|  |  | TNFα | down | 0.011 |

* Nineteen cytokines were evaluated (see Figure 4 for list of cytokines and the relative differences for each)

** *P* value of all other tissue cytokines were > 0.05.
