# Supplementary material for: Resurgence of Persisting Non-Cultivable Borrelia burgdorferi following Antibiotic Treatment in Mice
Source: PLoS One. 2014 Jan 23;9(1):e86907. doi: 10.1371/journal.pone.0086907 (PMC3900665; doi:10.1371/journal.pone.0086907)
Supplement: Table S3 — LDA analysis for RNA transcription of selected genes located throughout the B. burgdorferi cN40 genome in flaB DNA-positive heart base (HB) and tibiotarsal (Tt) tissue samples from infected mice at 12 months following treatment with saline or antibiotic. (DOCX) [file pone.0086907.s003.docx]

**Table S3.** LDA analysis for RNA transcription of selected genes located throughout the *B.* *burgdorferi* cN40 genome in *flaB* DNA-positive heart base (HB) and tibiotarsal (Tt) tissue samples from infected mice at 12 months following treatment with saline or antibiotic.

|  |  | **Saline** | | **Antibiotic** | |
| --- | --- | --- | --- | --- | --- |
| **Gene** | **Location** | **HB** | **Tt** | **HB** | **Tt** |
| 16S | ribosome | 5/5 | 5/5 | 3/3 | 7/7 |
| *bgp* | chromosome | 4/5 | 0/5 | 1/3 | 2/7 |
| *bmpA* |  | 4/5 | 0/5 | 1/3 | 1/7 |
| *bmpB* |  | 4/5 | 0/5 | 0/3 | 0/7 |
| *bmpC* |  | 3/5 | 0/5 | 1/3 | 2/7 |
| *flaB* |  | 5/5 | 0/5 | 1/3 | 3/7 |
| *ftsZ* |  | 4/5 | 0/5 | 0/3 | 1/7 |
| *gapDH* |  | 5/5 | 0/5 | 0/3 | 1/7 |
| *mtrC* |  | 3/5 | 0/5 | 0/3 | 0/7 |
| *oppA* |  | 3/5 | 1/5 | 2/3 | 1/7 |
| *P13* |  | 5/5 | 1/5 | 2/3 | 3/7 |
| *P66* |  | 3/5 | 0/5 | 0/3 | 0/7 |
| *recA* |  | 5/5 | 0/5 | 0/3 | 0/7 |
| *dbpA* | lp54 | 5/5 | 1/5 | 2/3 | 2/7 |
| *bbk32* | lp36 | 5/5 | 0/5 | 2/3 | 4/7 |
| *arp* | lp28-5 | 5/5 | 1/5 | 2/3 | 3/7 |
| *erp23* | cp32-4 | 3/5 | 0/5 | 0/3 | 1/7 |
| *erp24* | cp32-4 | 4/5 | 1/5 | 0/3 | 2/7 |
| *erp25* | cp32-5 | 3/5 | 0/5 | 0/3 | 2/7 |
| *ospE* | cp32-7 | 3/5 | 1/5 | 1/3 | 2/7 |
| *erp26* | cp32-10 | 3/5 | 1/5 | 1/3 | 1/7 |
| *erp27* | cp32-12 | 4/5 | 0/5 | 2/3 | 1/7 |

Transcription of the following genes were negative in both groups of mice, including chromosomal genes: *ackA*, *acrB, alr, bmpD, colV, glpA, HP, oms38, relA, s2lip, tgt, rrp1:* lp54 genes *CRASP1, ospA;* lp25 gene *bptA;* lp28-1 gene *vlsE;* cp32-9 gene *erp22;* cp26 gene *ospC;* cp9 gene *eppA;* and gene *p23T2* (unknown location)
